# Bis(di-2-pyridylmethane­diol-κ^3^ *N*,*O*,*N*′)copper(II) dl-tartrate 

**DOI:** 10.1107/S1600536808034983

**Published:** 2008-11-08

**Authors:** Jun Zhao, Dong-Sheng Li, Wen-Wen Dong, Dan-Jun Wang, Li Guo

**Affiliations:** a College of Mechanical & Materials Engineering, Three Gorges University, Yichang 443002, People’s Republic of China; b Department of Chemistry and Chemical Engineering, Shaanxi Key Laboratory of Chemical Reaction Engineering, Yan’an University, Yan’an 716000, People’s Republic of China

## Abstract

The reaction of di-2-pyridyl ketone with copper dichloride dihydrate and tartaric acid in water afforded the title compound, [Cu(C_11_H_10_N_2_O_2_)_2_]C_4_H_4_O_6_. The Cu^II^ atom lies on an inversion center *N*,*O*,*N*′-chelated by two di-2-pyridylmethane­diol ligands in a tetragonally distorted octa­hedral geometry. The tartrate anion is also located on an inversion center and has disordered hydroxyl groups, each with an occupancy factor of 0.5. The hydroxyl groups of the complex cation are hydrogen bonded to the carboxyl­ate groups of the anion, thus connecting the two building units.

## Related literature

For backgroung on di-2-pyridylketone complexes, see: Deveson *et al.* (1996 ▶); Sommerer *et al.* (1993 ▶); Wang *et al.* (1986 ▶).
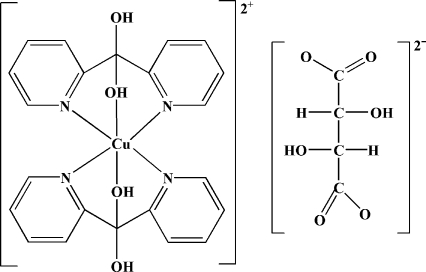


## Experimental

### 

#### Crystal data

[Cu(C_11_H_10_N_2_O_2_)_2_]C_4_H_4_O_6_
*M* *_r_* = 616.03
Triclinic, 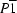

*a* = 7.7893 (8) Å
*b* = 8.1068 (8) Å
*c* = 11.3136 (12) Å
α = 105.973 (1)°
β = 90.431 (1)°
γ = 110.584 (1)°
*V* = 638.65 (11) Å^3^
*Z* = 1
Mo *K*α radiation
μ = 0.92 mm^−1^
*T* = 293 (2) K
0.45 × 0.30 × 0.18 mm

#### Data collection

Bruker SMART APEX CCD area-detector diffractometer
Absorption correction: multi-scan (*SADABS*; Sheldrick, 1996 ▶) *T*_min_ = 0.726, *T*_max_ = 0.850
3231 measured reflections
2235 independent reflections
1978 reflections with *I* > 2σ(*I*)
*R*_int_ = 0.015

#### Refinement

*R*[*F* ^2^ > 2σ(*F* ^2^)] = 0.038
*wR*(*F* ^2^) = 0.091
*S* = 1.03
2235 reflections
196 parameters
H-atom parameters constrained
Δρ_max_ = 0.37 e Å^−3^
Δρ_min_ = −0.31 e Å^−3^

### 

Data collection: *SMART* (Bruker, 2007 ▶); cell refinement: *SAINT* (Bruker, 2007 ▶); data reduction: *SAINT*; program(s) used to solve structure: *SHELXS97* (Sheldrick, 2008 ▶); program(s) used to refine structure: *SHELXL97* (Sheldrick, 2008 ▶); molecular graphics: *SHELXTL* (Sheldrick, 2008 ▶); software used to prepare material for publication: *SHELXTL*.

## Supplementary Material

Crystal structure: contains datablocks I, global. DOI: 10.1107/S1600536808034983/hy2156sup1.cif

Structure factors: contains datablocks I. DOI: 10.1107/S1600536808034983/hy2156Isup2.hkl

Additional supplementary materials:  crystallographic information; 3D view; checkCIF report

## Figures and Tables

**Table:** 

Cu1—N1	2.003 (2)
Cu1—N2	2.019 (2)
Cu1—O1	2.3920 (19)

**Table:** 

N1—Cu1—N2^i^	91.08 (9)
N1—Cu1—N2	88.92 (9)
N1—Cu1—O1^i^	104.11 (8)
N2—Cu1—O1^i^	106.37 (8)
N1—Cu1—O1	75.89 (8)
N2—Cu1—O1	73.63 (8)

Symmetry code: (i) 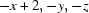.

**Table 2:** Hydrogen-bond geometry (Å, °)

*D*—H⋯*A*	*D*—H	H⋯*A*	*D*⋯*A*	*D*—H⋯*A*
O1—H1*A*⋯O3^ii^	0.85	1.73	2.582 (3)	178
O2—H2*A*⋯O4^ii^	0.82	1.84	2.648 (3)	170
O5—H5⋯O3	0.82	2.15	2.641 (5)	119
O6—H6⋯O4	0.82	2.22	2.693 (5)	118
C2—H2⋯O5^iii^	0.93	2.38	3.249 (6)	156
C3—H3⋯O4^iv^	0.93	2.50	3.217 (4)	134
C4—H4⋯O5	0.93	2.45	3.258 (5)	146

Symmetry codes: (ii) 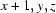; (iii) 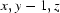; (iv) 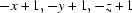.

**Fig. 1.**
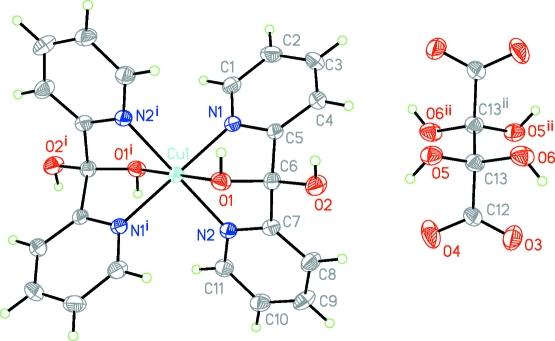
The structure of the title compound. Displacement ellipsoids are drawn at the 30% probability level. The hydroxyl groups (O5 and O6) of the tartrate anion are half-occupied. The disordered H atoms attached to C13 have been omitted. [Symmetry codes: (i) 2 - x, -y, -z; (ii) 1 - x, 1 - y, 1 - z.]

## supplementary crystallographic information

### Comment

Di-2-pyridylketone (dpk) functions either as a bidentate N,*N*'-donor or as a tridentate N,*O*,*N*'-donor towards metal ions, depending on the reaction medium used in the synthesis of the complexes (Deveson *et al.*, 1996), and several mononuclear and polynuclear transition metal–dpk complexes have been reported (Sommerer *et al.*, 1993; Wang *et al.*, 1986). The structural investigations clearly demonstrate that in each case hydration occurs across the ketone double bond in the ligand and that the resulting hydroxyl group coordinates to metal.

In the title compound, two dipyridin-2-yl-methanediol ligands, each in a tridentate fashion, are bonded to the Cu^II^ atom lying on an inversion center (Fig. 1). The pyridyl N atoms are strongly coordinated to the metal in the equatorial plane, while the hydroxyl groups are relatively weakly coordinated in the axial positions (Table 1). The two Cu—*O*(hydroxy) bonds [2.392 (2) Å], being in a *trans* arrangement, significantly exceed the Cu—N bond distances, a feature which can be attributed to the Jahn-Teller effect and usually manifests in *d*^9^ metal systems. The tartrate anion is located on an inversion center with disordered hydroxyl groups, each has an occupancy factor of 0.5. The hydroxyl groups of the complex cation as donors are involved in hydrogen bonds with the tartrate anion (Table 2).

### Experimental

A mixture of di-2-pyridylketone (0.184 g, 1 mmol), CuCl_2_.2H_2_O (0.067 g, 0.5 mmol), tartaric acid (0.075 g, 0.5 mmol) and water (18 ml) in a 25 ml Teflon-lined stainless steel reactor was heated from 298 to 453 K in 2 h and maintained at 453 K for 72 h. After the mixture was cooled to 298 K, blue crystals of the title compound were obtained.

### Refinement

All H atoms were positioned geometrically. Aromatic H atoms were refined as riding atoms, with C—H = 0.93 Å and *U*_iso_(H) = 1.2*U*_eq_(C). The other H atoms were fixed in the refinements, with *U*_iso_(H) = 1.2*U*_eq_(C,*O*).

### Crystal data

**Table:**

[Cu(C_11_H_10_N_2_O_2_)_2_]C_4_H_4_O_6_	*Z* = 1
*M**_r_* = 616.03	*F*_000_ = 317
Triclinic, *P*1	*D*_x_ = 1.602 Mg m^−^^3^
Hall symbol: -P 1	Mo *K*α radiation λ = 0.71073 Å
*a* = 7.7893 (8) Å	Cell parameters from 1352 reflections
*b* = 8.1068 (8) Å	θ = 2.8–26.5º
*c* = 11.3136 (12) Å	µ = 0.92 mm^−^^1^
α = 105.973 (1)º	*T* = 293 (2) K
β = 90.431 (1)º	Prism, blue
γ = 110.584 (1)º	0.45 × 0.30 × 0.18 mm
*V* = 638.65 (11) Å^3^	

### Data collection

**Table:**

Bruker SMART APEX CCD area-detector diffractometer	2235 independent reflections
Radiation source: fine-focus sealed tube	1978 reflections with *I* > 2σ(*I*)
Monochromator: graphite	*R*_int_ = 0.015
*T* = 293(2) K	θ_max_ = 25.1º
φ and ω scans	θ_min_ = 1.9º
Absorption correction: multi-scan(SADABS; Sheldrick, 1996)	*h* = −9→9
*T*_min_ = 0.726, *T*_max_ = 0.850	*k* = −9→8
3231 measured reflections	*l* = −13→11

### Refinement

**Table:**

Refinement on *F*^2^	Secondary atom site location: difference Fourier map
Least-squares matrix: full	Hydrogen site location: inferred from neighbouring sites
*R*[*F*^2^ > 2σ(*F*^2^)] = 0.038	H-atom parameters constrained
*wR*(*F*^2^) = 0.091	*w* = 1/[σ^2^(*F*_o_^2^) + (0.0346*P*)^2^ + 0.5737*P*] where *P* = (*F*_o_^2^ + 2*F*_c_^2^)/3
*S* = 1.04	(Δ/σ)_max_ < 0.001
2235 reflections	Δρ_max_ = 0.37 e Å^−^^3^
196 parameters	Δρ_min_ = −0.31 e Å^−^^3^
Primary atom site location: structure-invariant direct methods	Extinction correction: none

### Special details

**Table:**

Geometry. All e.s.d.'s (except the e.s.d. in the dihedral angle between two l.s. planes) are estimated using the full covariance matrix. The cell e.s.d.'s are taken into account individually in the estimation of e.s.d.'s in distances, angles and torsion angles; correlations between e.s.d.'s in cell parameters are only used when they are defined by crystal symmetry. An approximate (isotropic) treatment of cell e.s.d.'s is used for estimating e.s.d.'s involving l.s. planes.

### Fractional atomic coordinates and isotropic or equivalent isotropic displacement parameters (Å^2^)

**Table:**

	*x*	*y*	*z*	*U*_iso_*/*U*_eq_	Occ. (<1)
Cu1	1.0000	0.0000	0.0000	0.03430 (17)	
N1	0.9447 (3)	0.0410 (3)	0.1761 (2)	0.0337 (5)	
N2	0.8085 (3)	0.1020 (3)	−0.0329 (2)	0.0357 (5)	
O1	1.1369 (3)	0.3273 (3)	0.09003 (17)	0.0394 (5)	
H1A	1.2267	0.3677	0.1473	0.047*	
O2	1.0017 (3)	0.5166 (3)	0.21831 (19)	0.0488 (6)	
H2A	1.0963	0.5631	0.2671	0.059*	
O3	0.4119 (4)	0.4426 (5)	0.2591 (2)	0.0951 (11)	
O4	0.2982 (5)	0.6254 (5)	0.3782 (3)	0.1005 (13)	
O5	0.7064 (6)	0.5609 (6)	0.4214 (4)	0.0507 (11)	0.50
H5	0.6847	0.5143	0.3468	0.061*	0.50
O6	0.5874 (7)	0.7540 (6)	0.5538 (4)	0.0569 (12)	0.50
H6	0.5146	0.7973	0.5366	0.068*	0.50
C1	0.9098 (4)	−0.0858 (4)	0.2372 (3)	0.0411 (7)	
H1	0.9154	−0.2004	0.1977	0.049*	
C2	0.8658 (5)	−0.0499 (5)	0.3569 (3)	0.0506 (8)	
H2	0.8416	−0.1391	0.3979	0.061*	
C3	0.8583 (5)	0.1207 (5)	0.4150 (3)	0.0538 (9)	
H3	0.8274	0.1468	0.4955	0.065*	
C4	0.8970 (4)	0.2526 (5)	0.3533 (3)	0.0459 (8)	
H4	0.8936	0.3685	0.3915	0.055*	
C5	0.9407 (4)	0.2086 (4)	0.2340 (2)	0.0339 (6)	
C6	0.9820 (4)	0.3391 (4)	0.1516 (3)	0.0361 (6)	
C7	0.8205 (4)	0.2618 (4)	0.0498 (3)	0.0365 (6)	
C8	0.6957 (4)	0.3441 (4)	0.0418 (3)	0.0472 (8)	
H8	0.7037	0.4522	0.1017	0.057*	
C9	0.5585 (4)	0.2637 (5)	−0.0563 (3)	0.0540 (9)	
H9	0.4729	0.3174	−0.0638	0.065*	
C10	0.5493 (4)	0.1036 (5)	−0.1432 (3)	0.0483 (8)	
H10	0.4593	0.0492	−0.2111	0.058*	
C11	0.6747 (4)	0.0247 (4)	−0.1282 (3)	0.0420 (7)	
H11	0.6665	−0.0854	−0.1858	0.050*	
C12	0.4060 (4)	0.5461 (4)	0.3591 (3)	0.0436 (7)	
C13	0.5422 (4)	0.5732 (4)	0.4673 (3)	0.0406 (7)	
H13A	0.5713	0.6994	0.5271	0.049*	0.50
H13B	0.6568	0.5597	0.4349	0.049*	0.50

### Atomic displacement parameters (Å^2^)

**Table:**

	*U*^11^	*U*^22^	*U*^33^	*U*^12^	*U*^13^	*U*^23^
Cu1	0.0397 (3)	0.0388 (3)	0.0277 (3)	0.0214 (2)	0.0000 (2)	0.0060 (2)
N1	0.0388 (13)	0.0359 (13)	0.0302 (12)	0.0188 (11)	0.0011 (10)	0.0092 (10)
N2	0.0385 (13)	0.0404 (13)	0.0289 (12)	0.0186 (11)	−0.0017 (10)	0.0063 (10)
O1	0.0425 (11)	0.0413 (11)	0.0322 (10)	0.0143 (9)	−0.0019 (9)	0.0090 (9)
O2	0.0607 (14)	0.0365 (12)	0.0458 (13)	0.0235 (10)	−0.0110 (11)	0.0002 (9)
O3	0.0785 (19)	0.152 (3)	0.0442 (15)	0.076 (2)	−0.0195 (14)	−0.0292 (17)
O4	0.130 (3)	0.121 (3)	0.0594 (18)	0.100 (2)	−0.0409 (17)	−0.0290 (17)
O5	0.041 (2)	0.062 (3)	0.051 (3)	0.019 (2)	0.001 (2)	0.019 (2)
O6	0.068 (3)	0.043 (3)	0.047 (3)	0.011 (2)	−0.012 (2)	0.006 (2)
C1	0.0428 (17)	0.0410 (17)	0.0433 (17)	0.0191 (14)	0.0003 (14)	0.0136 (14)
C2	0.053 (2)	0.062 (2)	0.0462 (19)	0.0234 (17)	0.0046 (15)	0.0292 (17)
C3	0.062 (2)	0.078 (2)	0.0307 (16)	0.0362 (19)	0.0097 (15)	0.0165 (17)
C4	0.0552 (19)	0.0545 (19)	0.0335 (16)	0.0310 (16)	0.0035 (14)	0.0077 (14)
C5	0.0353 (15)	0.0404 (16)	0.0290 (14)	0.0200 (13)	−0.0005 (12)	0.0070 (12)
C6	0.0447 (16)	0.0324 (15)	0.0325 (15)	0.0193 (13)	0.0014 (13)	0.0053 (12)
C7	0.0409 (16)	0.0378 (16)	0.0357 (16)	0.0178 (13)	0.0031 (13)	0.0143 (13)
C8	0.0534 (19)	0.0406 (17)	0.054 (2)	0.0253 (15)	0.0002 (16)	0.0138 (15)
C9	0.0451 (19)	0.058 (2)	0.069 (2)	0.0278 (17)	−0.0049 (17)	0.0218 (18)
C10	0.0428 (17)	0.057 (2)	0.0441 (18)	0.0197 (16)	−0.0083 (14)	0.0121 (16)
C11	0.0409 (17)	0.0451 (17)	0.0363 (16)	0.0159 (14)	−0.0037 (13)	0.0062 (13)
C12	0.0466 (18)	0.0448 (18)	0.0353 (17)	0.0162 (15)	0.0008 (14)	0.0062 (14)
C13	0.0396 (16)	0.0404 (17)	0.0375 (16)	0.0135 (13)	−0.0019 (13)	0.0066 (13)

### Geometric parameters (Å, °)

**Table:**

Cu1—N1^i^	2.003 (2)	C1—C2	1.377 (4)
Cu1—N1	2.003 (2)	C1—H1	0.9300
Cu1—N2^i^	2.019 (2)	C2—C3	1.380 (5)
Cu1—N2	2.019 (2)	C2—H2	0.9300
Cu1—O1^i^	2.3920 (19)	C3—C4	1.382 (5)
Cu1—O1	2.3920 (19)	C3—H3	0.9300
N1—C1	1.344 (4)	C4—C5	1.375 (4)
N1—C5	1.348 (3)	C4—H4	0.9300
N2—C11	1.339 (4)	C5—C6	1.549 (4)
N2—C7	1.345 (4)	C6—C7	1.526 (4)
O1—C6	1.417 (3)	C7—C8	1.373 (4)
O1—H1A	0.8554	C8—C9	1.376 (5)
O2—C6	1.382 (3)	C8—H8	0.9300
O2—H2A	0.8209	C9—C10	1.374 (5)
O3—C12	1.222 (4)	C9—H9	0.9300
O4—C12	1.213 (4)	C10—C11	1.374 (4)
O5—C13	1.409 (5)	C10—H10	0.9300
O5—H5	0.8134	C11—H11	0.9300
O5—H13B	0.4145	C12—C13	1.530 (4)
O6—C13	1.440 (5)	C13—C13^ii^	1.527 (6)
O6—H6	0.8117	C13—H13A	1.0044
O6—H13A	0.4359	C13—H13B	0.9970
			
N1^i^—Cu1—N1	180.0	N1—C5—C4	121.9 (3)
N1^i^—Cu1—N2^i^	88.92 (9)	N1—C5—C6	113.5 (2)
N1—Cu1—N2^i^	91.08 (9)	C4—C5—C6	124.6 (3)
N1^i^—Cu1—N2	91.08 (9)	O2—C6—O1	113.9 (2)
N1—Cu1—N2	88.92 (9)	O2—C6—C7	109.4 (2)
N2^i^—Cu1—N2	180.0	O1—C6—C7	105.5 (2)
N1^i^—Cu1—O1^i^	75.89 (8)	O2—C6—C5	111.9 (2)
N1—Cu1—O1^i^	104.11 (8)	O1—C6—C5	108.2 (2)
N2^i^—Cu1—O1^i^	73.63 (8)	C7—C6—C5	107.6 (2)
N2—Cu1—O1^i^	106.37 (8)	N2—C7—C8	122.0 (3)
N1^i^—Cu1—O1	104.11 (8)	N2—C7—C6	113.9 (2)
N1—Cu1—O1	75.89 (8)	C8—C7—C6	124.1 (3)
N2^i^—Cu1—O1	106.37 (8)	C7—C8—C9	118.8 (3)
N2—Cu1—O1	73.63 (8)	C7—C8—H8	120.6
O1^i^—Cu1—O1	180.00 (10)	C9—C8—H8	120.6
C1—N1—C5	119.4 (2)	C10—C9—C8	119.4 (3)
C1—N1—Cu1	124.79 (19)	C10—C9—H9	120.3
C5—N1—Cu1	115.84 (18)	C8—C9—H9	120.3
C11—N2—C7	118.8 (2)	C9—C10—C11	119.1 (3)
C11—N2—Cu1	125.7 (2)	C9—C10—H10	120.5
C7—N2—Cu1	115.45 (18)	C11—C10—H10	120.5
C6—O1—Cu1	93.97 (15)	N2—C11—C10	121.8 (3)
C6—O1—H1A	105.5	N2—C11—H11	119.1
Cu1—O1—H1A	116.8	C10—C11—H11	119.1
C6—O2—H2A	109.5	O4—C12—O3	124.4 (3)
C13—O5—H5	108.0	O4—C12—C13	118.5 (3)
C13—O5—H13B	5.1	O3—C12—C13	117.1 (3)
H5—O5—H13B	107.4	O5—C13—O6	107.9 (3)
C13—O6—H6	108.0	O5—C13—C12	108.8 (3)
C13—O6—H13A	2.7	O6—C13—C12	110.1 (3)
H6—O6—H13A	105.4	O5—C13—C13^ii^	110.6 (3)
N1—C1—C2	121.5 (3)	O6—C13—C13^ii^	109.6 (3)
N1—C1—H1	119.3	C12—C13—C13^ii^	109.9 (3)
C2—C1—H1	119.3	O5—C13—H13A	108.8
C1—C2—C3	119.0 (3)	O6—C13—H13A	1.2
C1—C2—H2	120.5	C12—C13—H13A	109.0
C3—C2—H2	120.5	C13^ii^—C13—H13A	109.7
C2—C3—C4	119.8 (3)	O5—C13—H13B	2.1
C2—C3—H3	120.1	O6—C13—H13B	109.5
C4—C3—H3	120.1	C12—C13—H13B	109.1
C5—C4—C3	118.5 (3)	C13^ii^—C13—H13B	108.6
C5—C4—H4	120.7	H13A—C13—H13B	110.5
C3—C4—H4	120.7		

Symmetry codes: (i) −*x*+2, −*y*, −*z*; (ii) −*x*+1, −*y*+1, −*z*+1.

### Hydrogen-bond geometry (Å, °)

**Table:**

*D*—H···*A*	*D*—H	H···*A*	*D*···*A*	*D*—H···*A*
O1—H1A···O3^iii^	0.85	1.73	2.582 (3)	178
O2—H2A···O4^iii^	0.82	1.84	2.648 (3)	170
O5—H5···O3	0.82	2.15	2.641 (5)	119
O6—H6···O4	0.82	2.22	2.693 (5)	118
C2—H2···O5^iv^	0.93	2.38	3.249 (6)	156
C3—H3···O4^ii^	0.93	2.50	3.217 (4)	134
C4—H4···O5	0.93	2.45	3.258 (5)	146

Symmetry codes: (iii) *x*+1, *y*, *z*; (iv) *x*, *y*−1, *z*; (ii) −*x*+1, −*y*+1, −*z*+1.
